# Supplementary figures and images for: Multiscale, multimodal analysis of tumor heterogeneity in IDH1 mutant vs wild-type diffuse gliomas
Source: PLoS One. 2019 Dec 27;14(12):e0219724. doi: 10.1371/journal.pone.0219724 (PMC6934292; doi:10.1371/journal.pone.0219724)

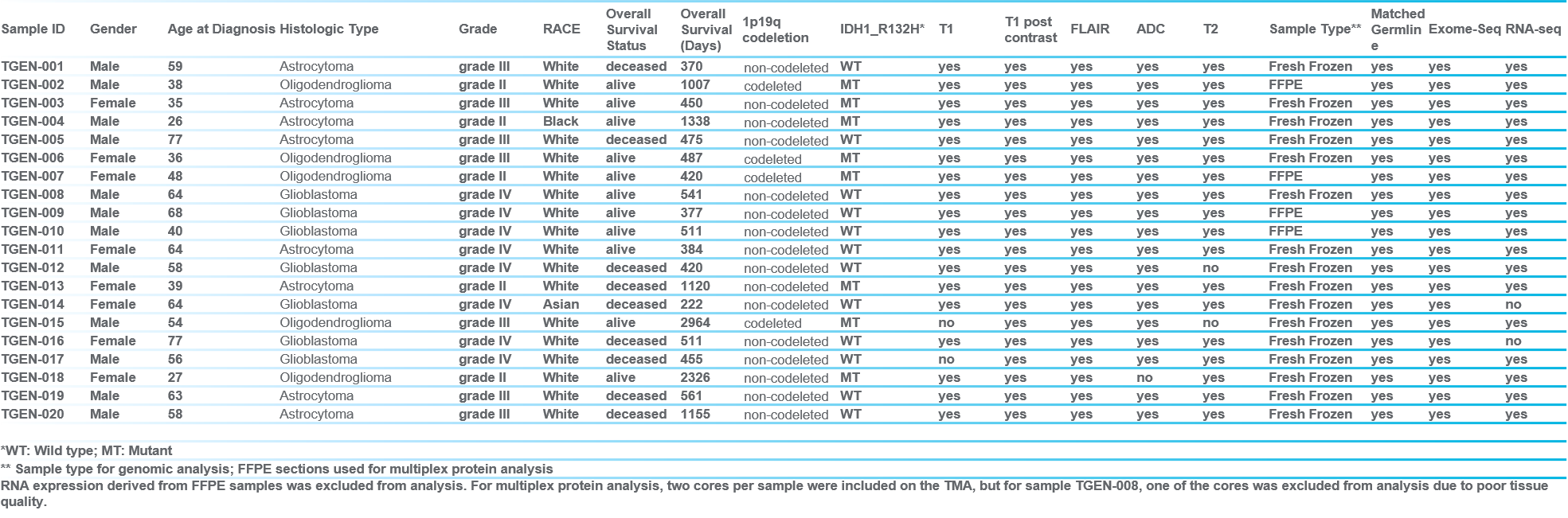

Supplement: S1 Table — (TIF) [file pone.0219724.s001.tif]

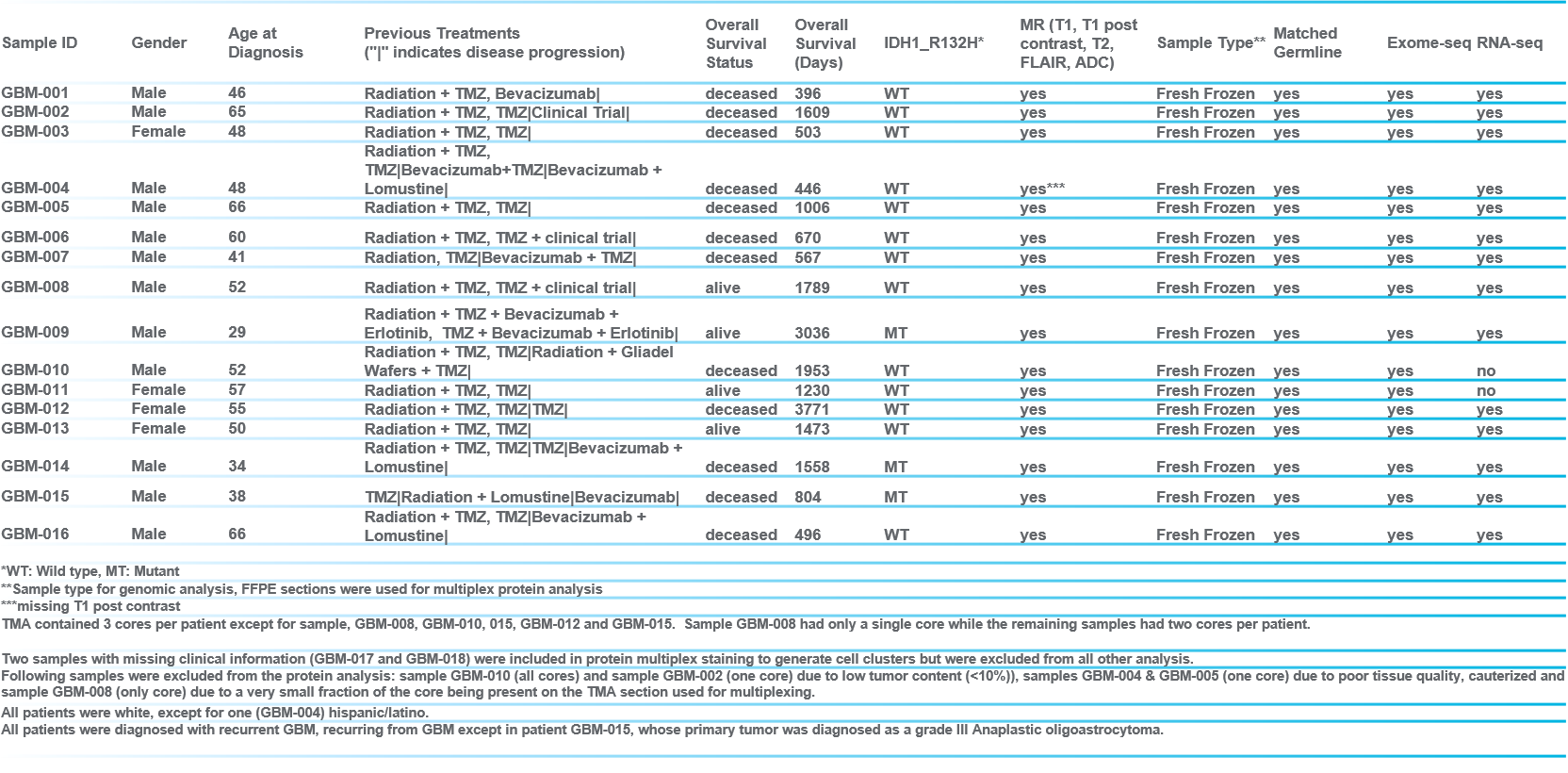

Supplement: S2 Table — (TIF) [file pone.0219724.s002.tif]

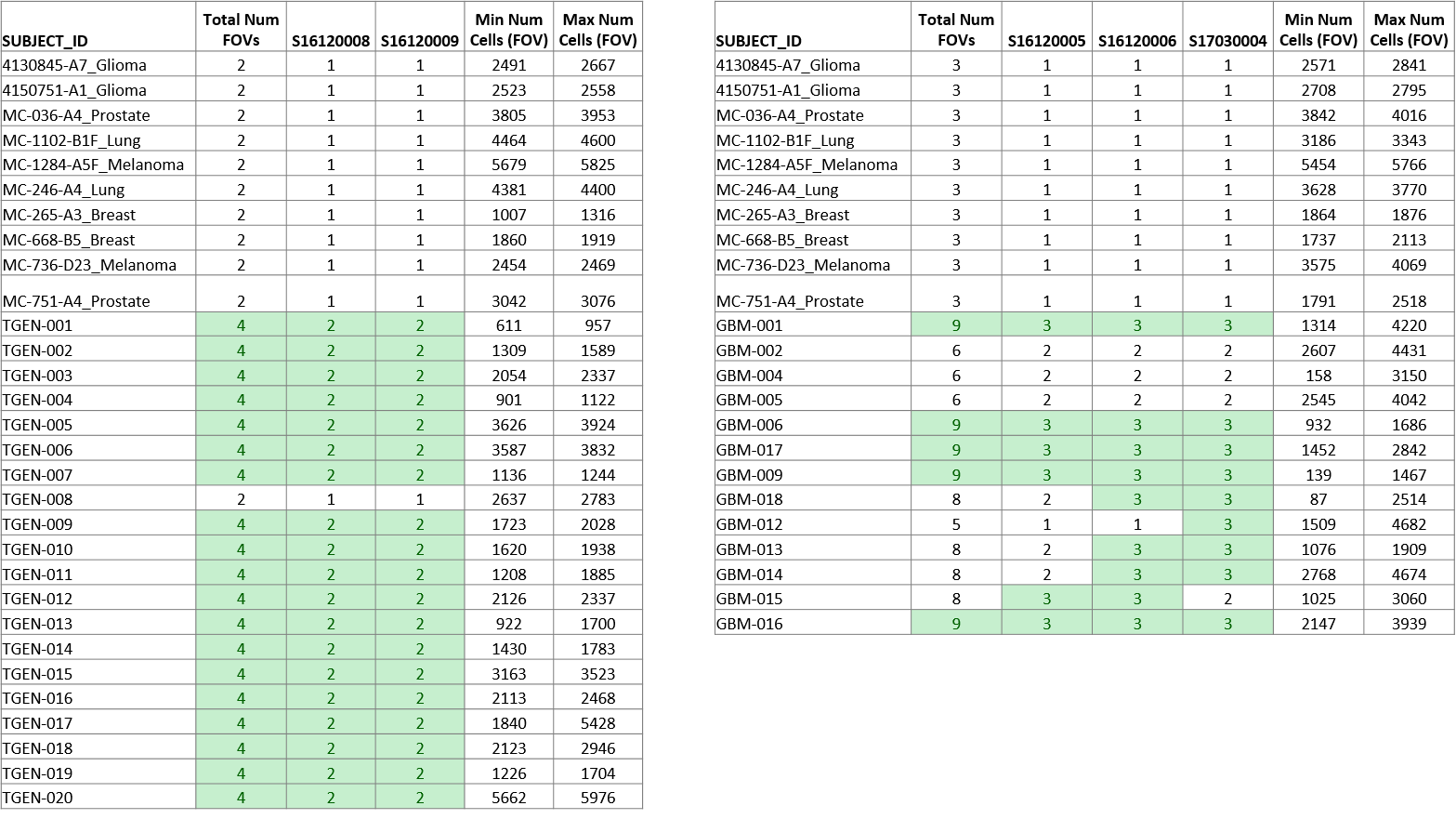

Supplement: S4 Table — (TIF) [file pone.0219724.s004.tif]

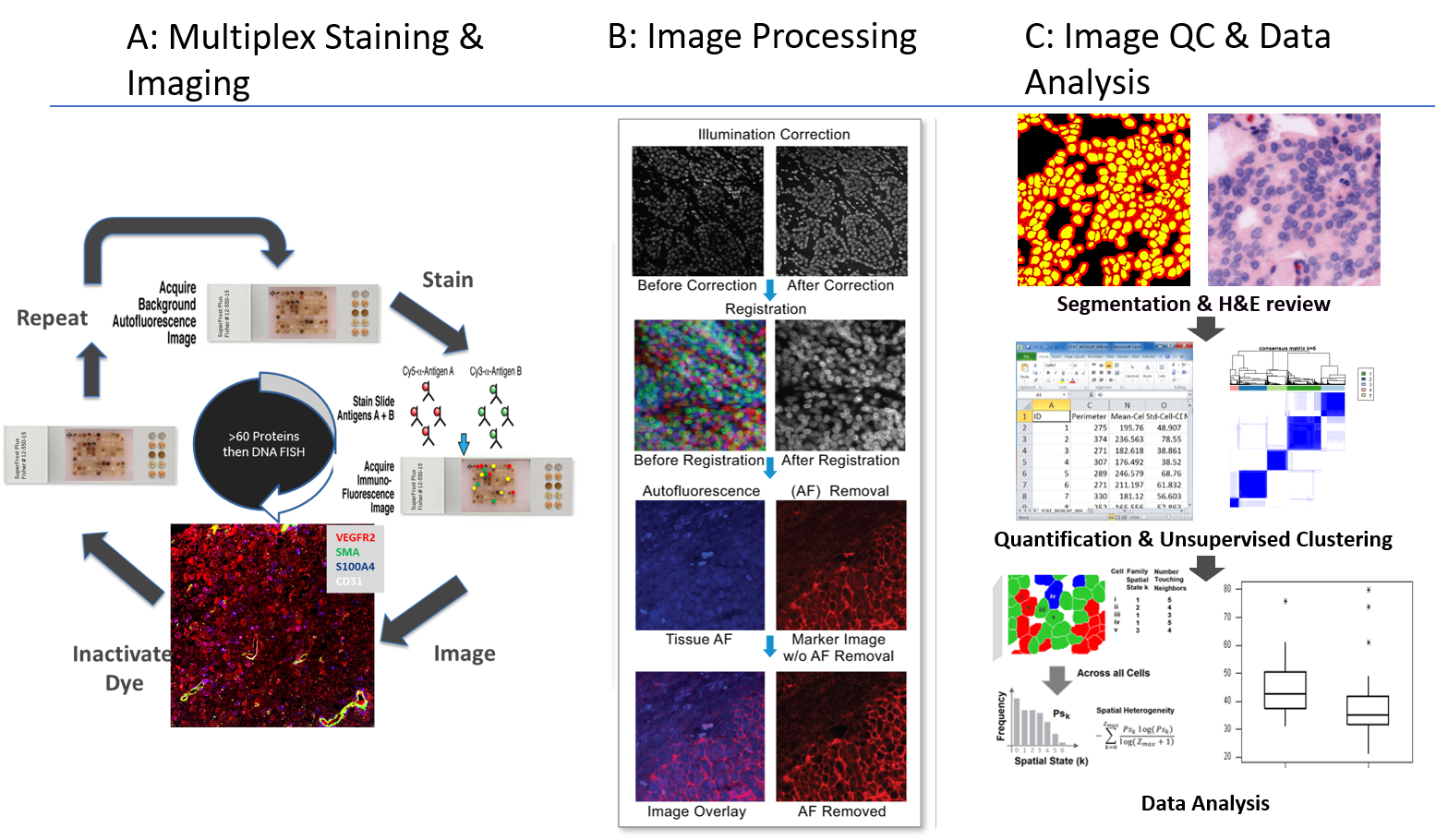

Supplement: S1 Fig — The Cell DIVE MxIF workflow involves repeated cycles of staining, imaging and signal inactivation (panel A), following slide clearing and antigen retrieval. Prior to antibody staining, tissue is stained with DAPI and imaged in all channels of interest to record background autofluorescence (AF) of the tissue. Following background imaging, tissue is stained with 2–3 antibodies and reimaged to capture antigen-specific signal and then undergoes a dye inactivation step to remove the signal. The slide is re-imaged to measure background fluorescence intensity. These cycles are repeated multiple times until all targets of interest have been imaged. Panel 2 shows various image processing steps prior to generating single cell data. Some of these are performed during imaging itself while others are performed post image acquisition. The steps include, illumination correction, to correct for uneven illumination across the FOV, registration of images from all rounds (using DAPI signal from each round) and tissue AF removal. Panel C: Staining intensity of various cellular and subcellular markers is used to generate cellular segmentation masks. Segmented images are compared with real or virtual H&Es (generated from DAPI stained background images at the beginning of multiplexing) by a trained biologist or pathologist, and images with poor segmentation are removed from analysis. In parallel, marker staining is evaluated by reviewing AF removed images and markers that failed to stain or images with large artefacts are removed from analysis. Marker expression is quantified at cellular and subcellular compartments and data is generated in an easy to use .csv or Excel format which is then analyzed by a variety of different tools/approaches including simple statistical correlations, cluster analysis as well as heterogeneity analysis. (TIF) [file pone.0219724.s005.tif]

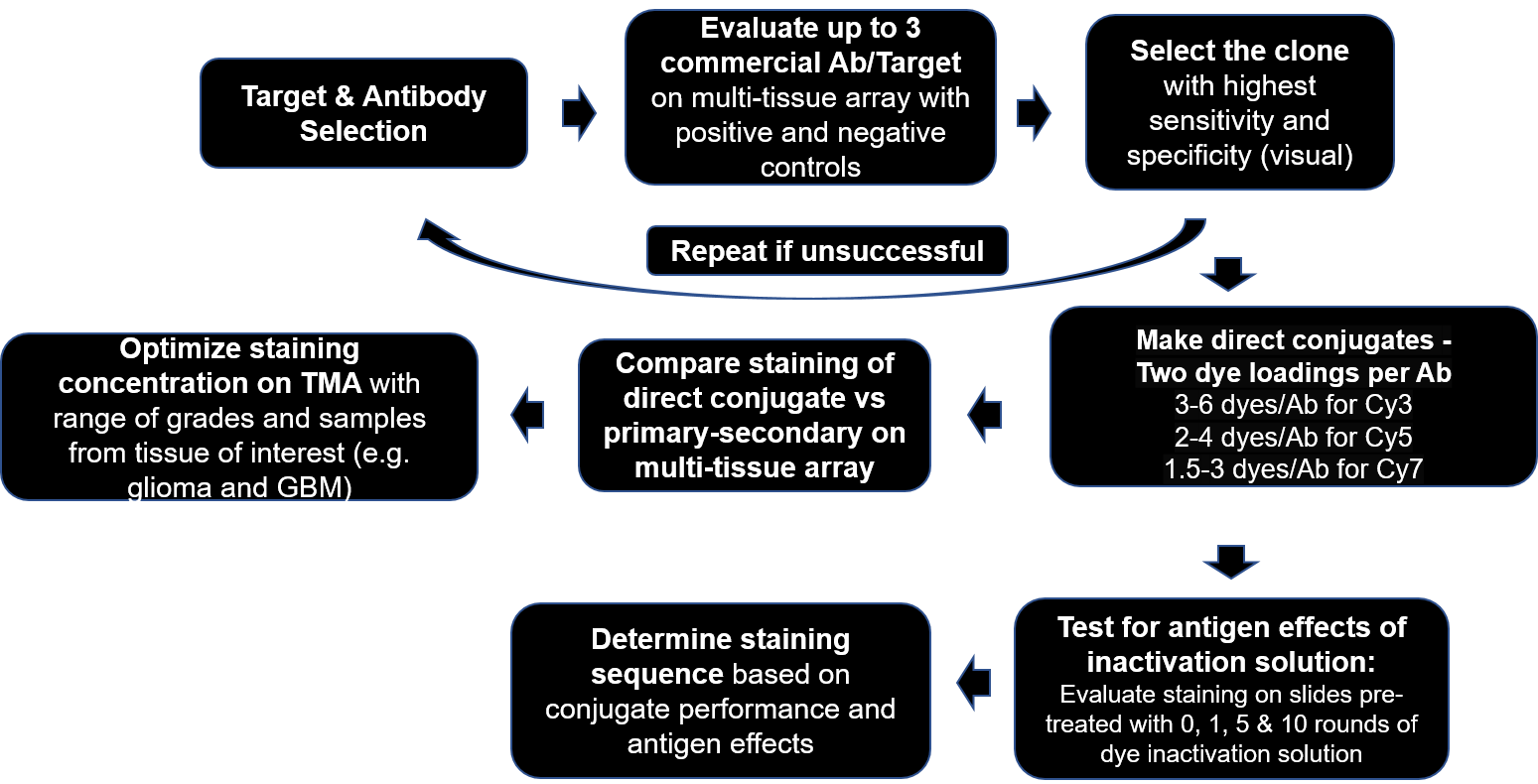

Supplement: S2 Fig — A typical antibody validation workflow: Starting with literature reports to identify antibody clones previously used for IHC on FFPE tissue, 3 or more clones per target are identified and evaluated for sensitivity and specificity of the signal on a multi-tissue array (TMA) comprising all major tumor types and corresponding normal tissues. The down-selected antibody is conjugated with CY3, Cy5 or Cy7 at 2 different dye/protein ratio and conjugates validated by staining comparison with unconjugated primary on serial sections of the same TMA. The down-selected conjugate is tested at different concentrations on a TMA with tumor tissue of interest to determine the optimal concentration for staining. In parallel, a set of TMA serial sections are pre-treated with different rounds of bleaching and evaluated for bleaching solution’s effect on antigen of interest by comparing the staining among this set. Antigens with discernible effects are prioritized for staining early in the sequence, immediately after primary secondary staining of targets which failed to conjugate. (TIF) [file pone.0219724.s006.tif]

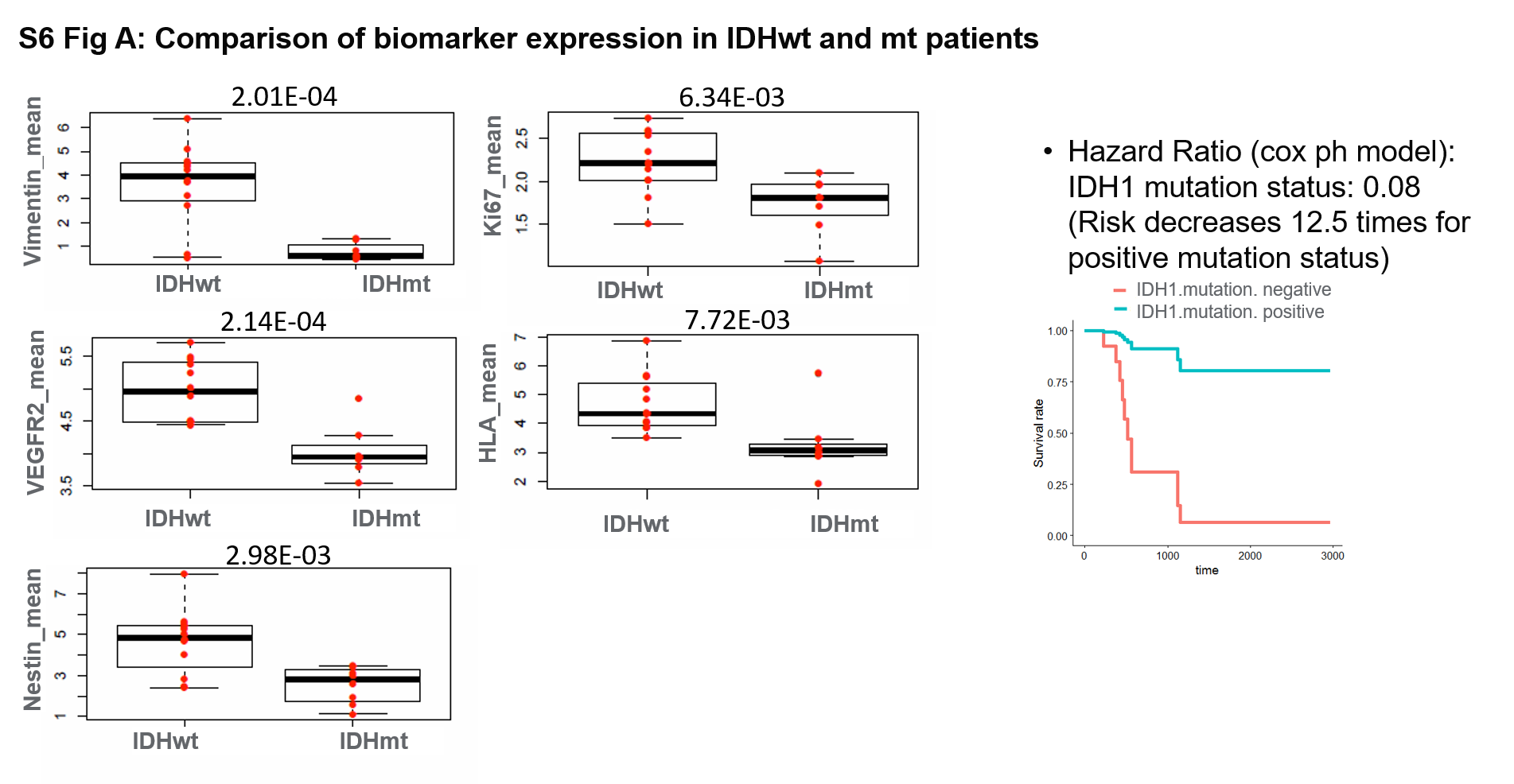

Supplement: S3 Fig — A: Marker staining performance in each cohort (True-positive, False-negative), staining round, subcellular location used for analysis and gene symbol, B: examples of quantitative FOV level correlation of marker intensities on replicate slides (Pearson correlation coefficients are shown), C: Examples of fluorescence image overlays of various hallmark markers showing heterogeneity of expression in astrocytoma. (TIFF) [file pone.0219724.s007.tiff]

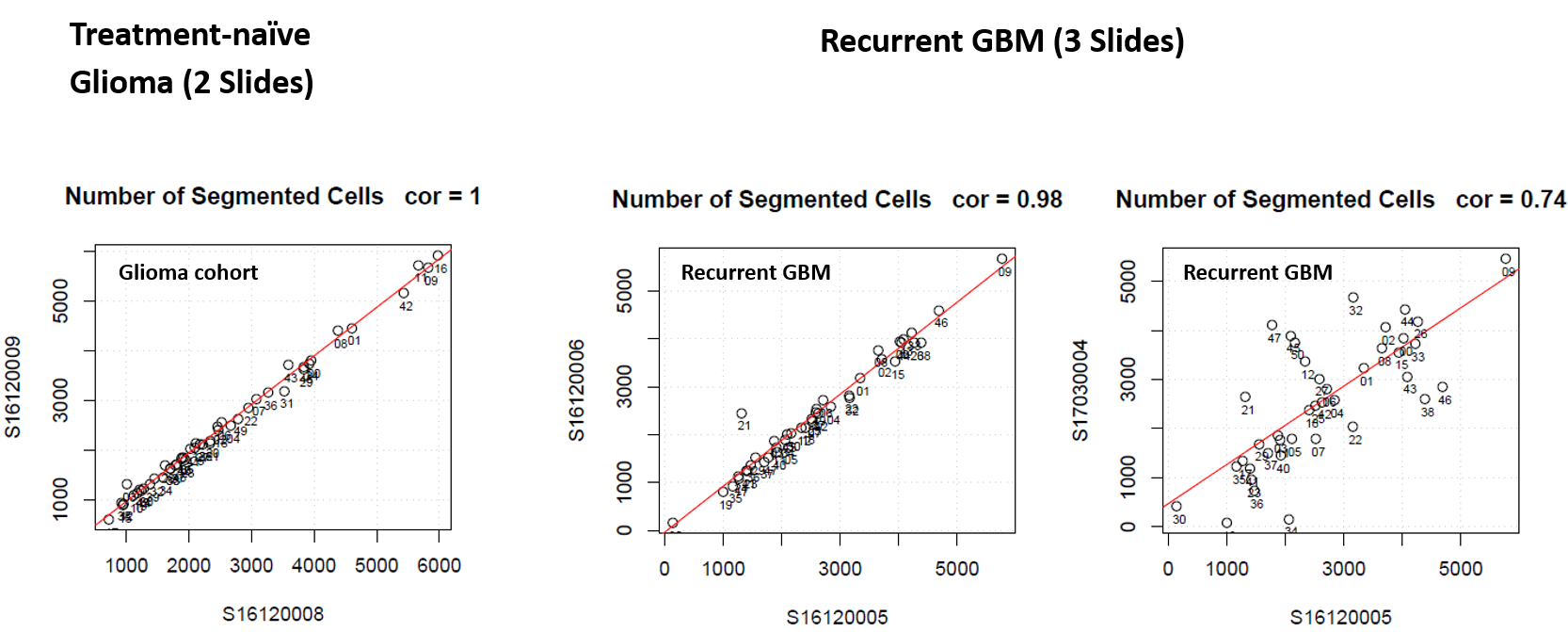

Supplement: S4 Fig — High correlation in number of segmented cells was observed between serial sections, particularly for the treatment naïve glioma cohort and two out of three sections of the recurrent GBM cohort. The Pearson correlation coefficient was computed and is presented in each slide to slide correlation plot. (TIF) [file pone.0219724.s008.tif]

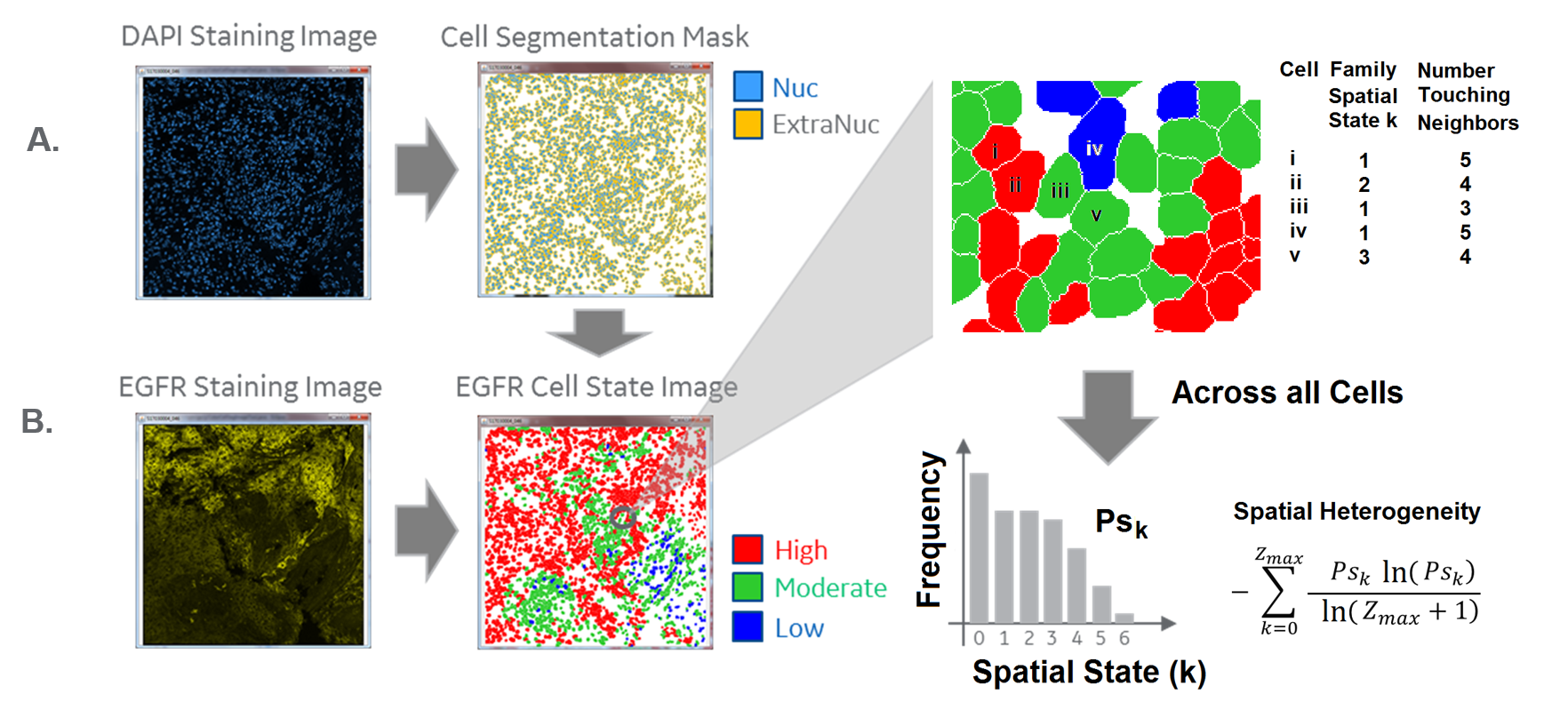

Supplement: S5 Fig — Example of how molecular state and cell spatial heterogeneity metrics are calculated, using EGFR as an example. A. Segmentation of cells using DAPI staining and generation of nuclear and extra-nuclear masks; B. EGFR fluorescence intensity is quantified for each cell and discretized as low, moderate, and high. The different levels of cell expression are shown as red (high), green (moderate) or blue (low). C. For each cell (I through v in this cartoon), adjacent neighboring (touching) cells are counted, and their Spatial State is used to sum the Spatial Heterogeneity. (TIF) [file pone.0219724.s009.tif]

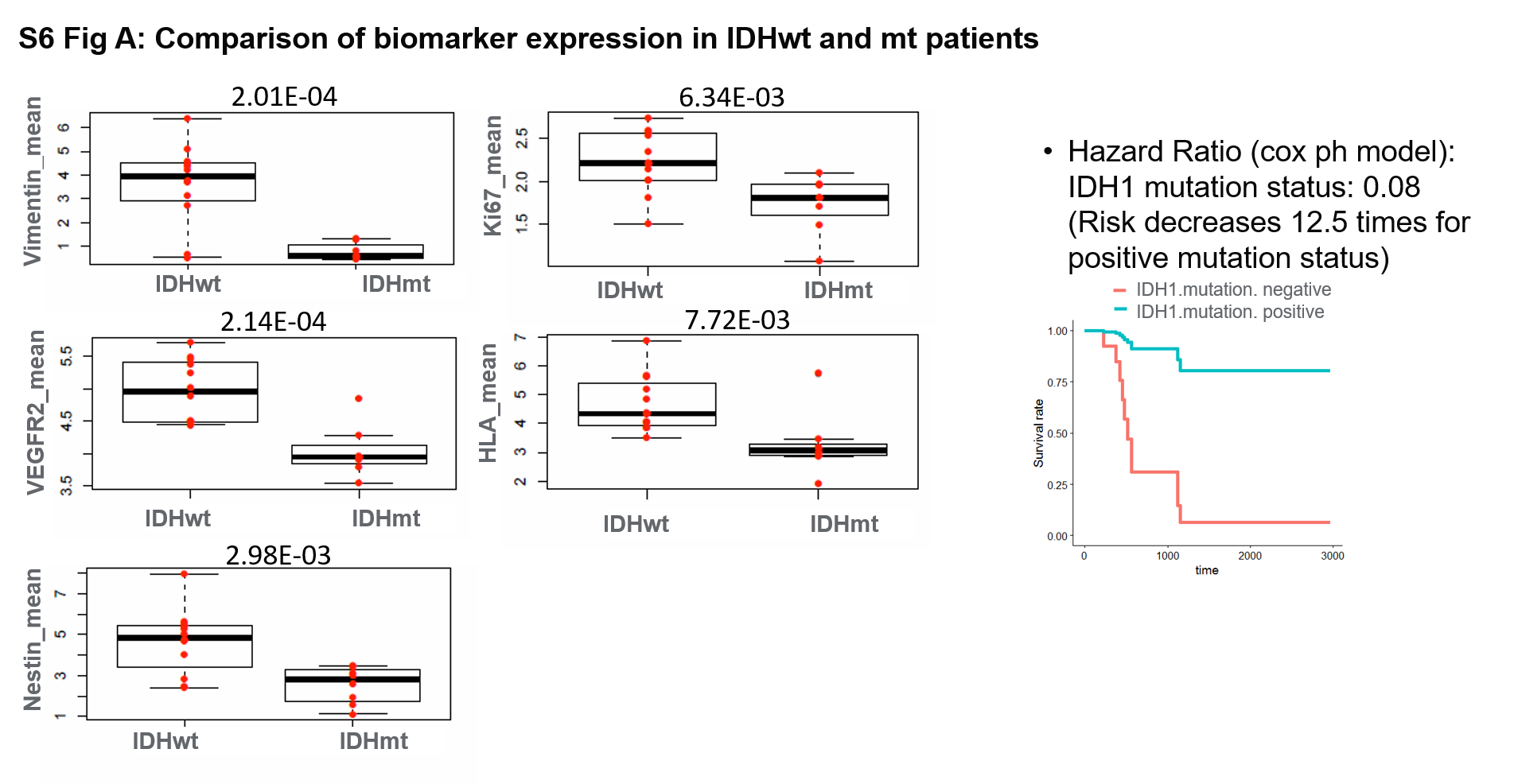

Supplement: S6 Fig — Uni- (A) and multivariate (B) analysis of biomarker expression and overall survival as a function of IDH mutation status A. Differences in individual biomarker expression and survival of IDHmt and IDHwt patients. B. A predictive multivariate model of IDH mutation status. (TIFF) [file pone.0219724.s010.tiff]

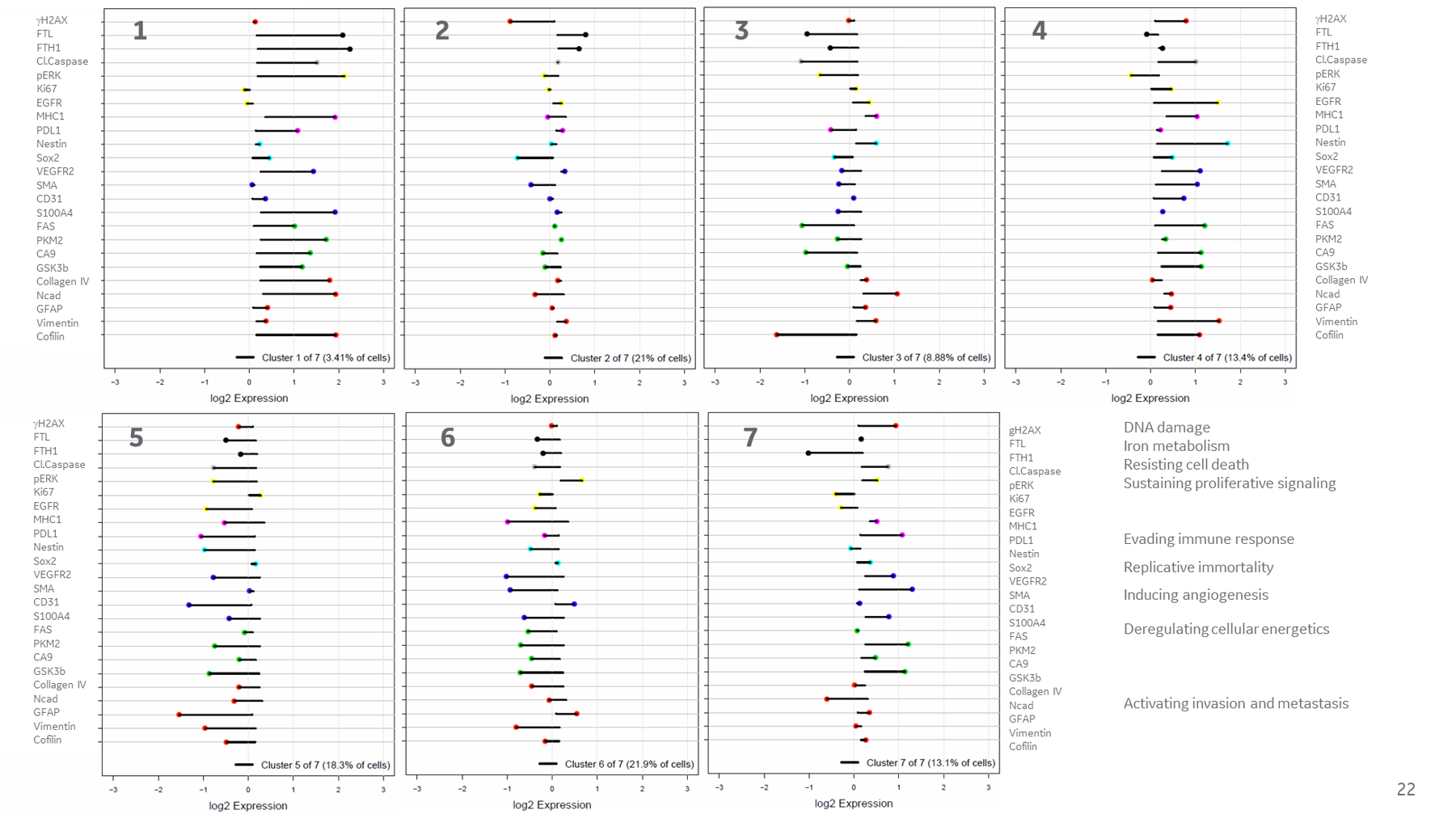

Supplement: S7 Fig — Protein expression profiles of individual clusters plotted relative to median expression in the whole population. Solid circles represent the average expression in the cluster while direction and length of the lollipop shows difference in expression relative to population median (left-lower, right-higher). (TIF) [file pone.0219724.s011.tif]

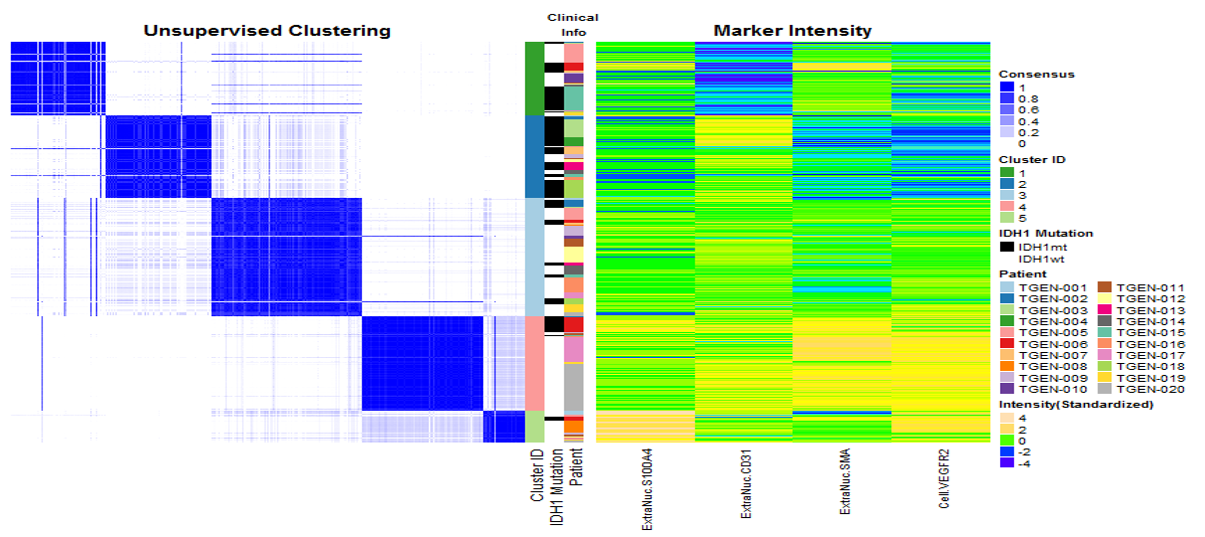

Supplement: S8 Fig — Unsupervised clustering of cells using angiogenesis hallmark proteins identified a 5 cluster set. Clusters with lower than average hallmark protein expression (1 & 2) are highly represented in samples with IDH1 mutation. Cluster 4 & 5 with higher expression are proportionally more abundant in IDH1wt samples. (TIF) [file pone.0219724.s012.tif]

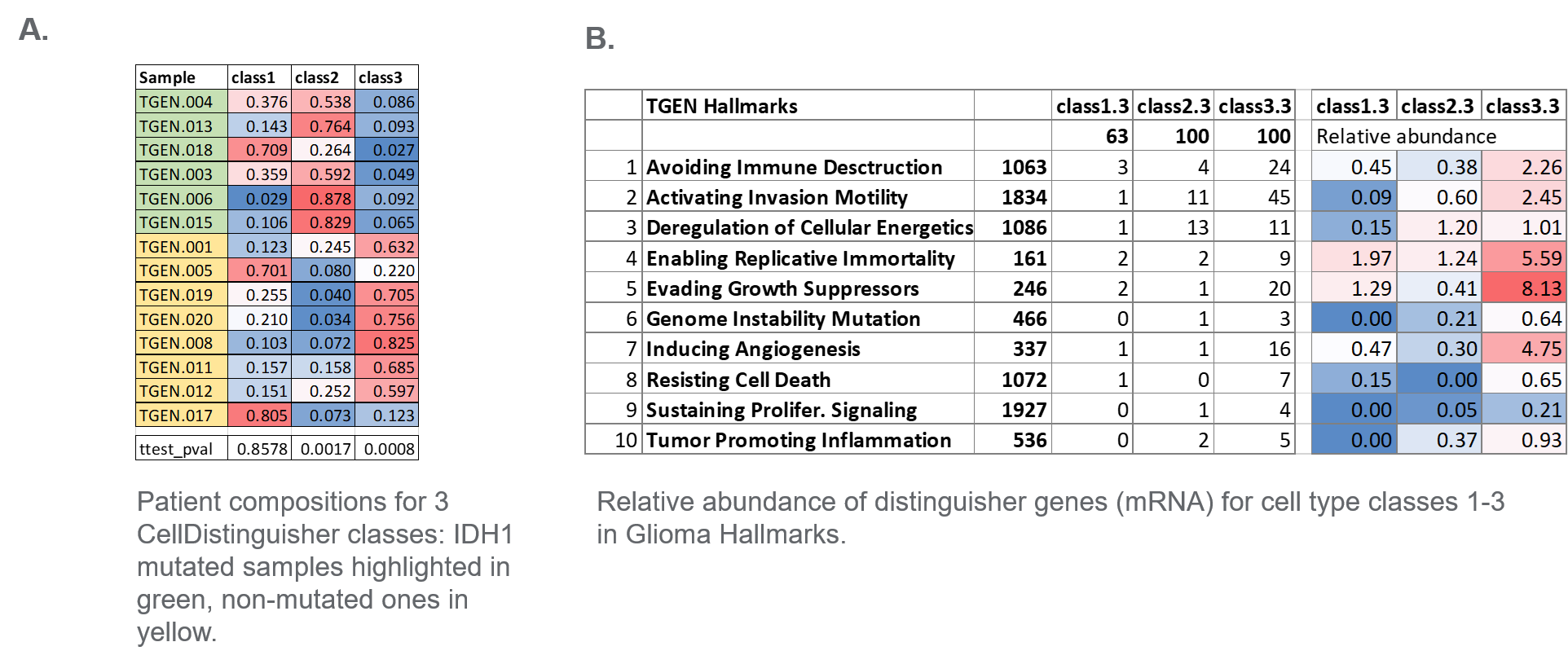

Supplement: S9 Fig — A: Relative proportion of cells belonging to different CellDistinguisher classes in each sample. Class 3 is highly represented in IDHwt samples. B: shows relative abundance of distinguisher genes grouped by hallmarks in individual classes. (TIF) [file pone.0219724.s013.tif]

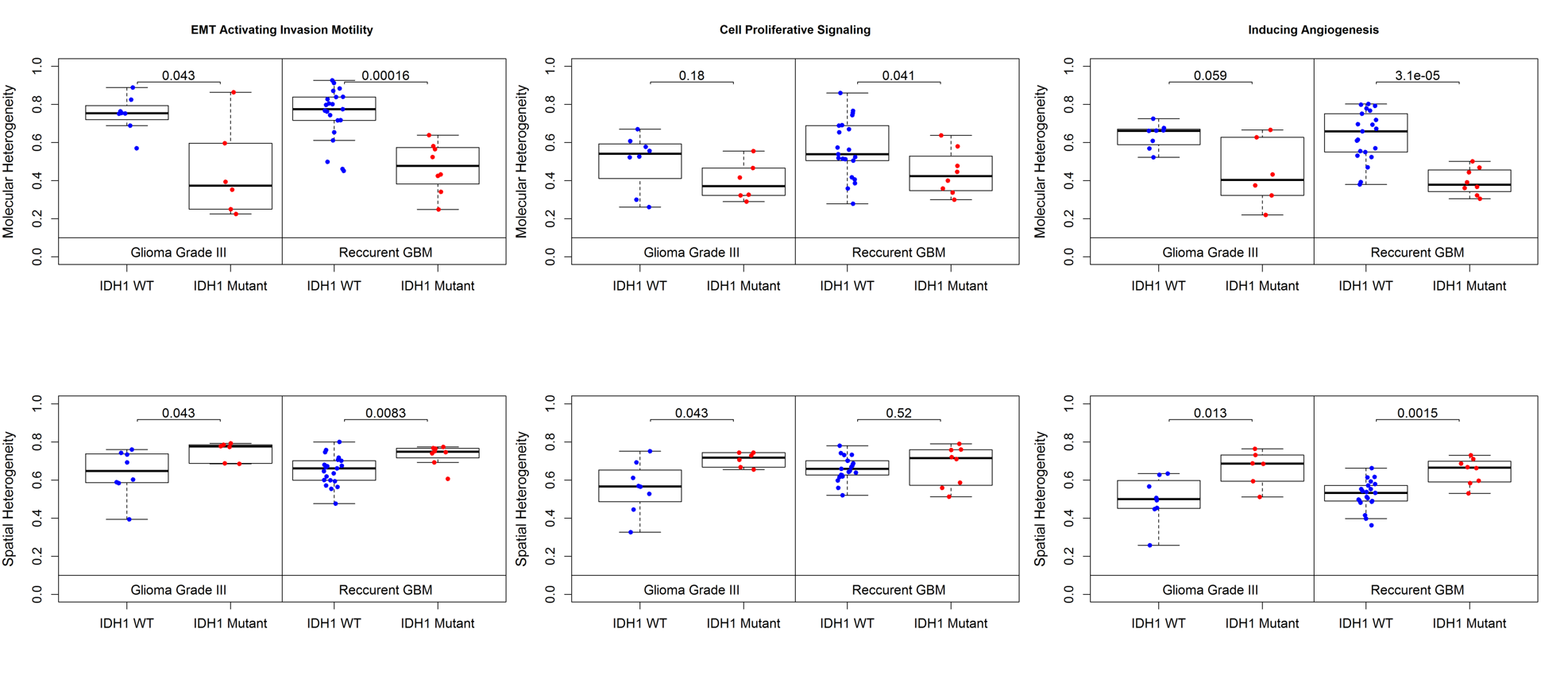

Supplement: S10 Fig — Molecular and spatial heterogeneity in grade III gliomas and recurrent GBM IDHwt and IDHmt tumors according to the following hallmarks: Invasion and Motility, Cell Proliferative Signaling and Inducing Angiogenesis. The Mann-Whitney test was performed on all unpaired comparisons with the resulting p-value presented in each plot. The p-values were not adjusted for multiple testing. (TIF) [file pone.0219724.s014.tif]

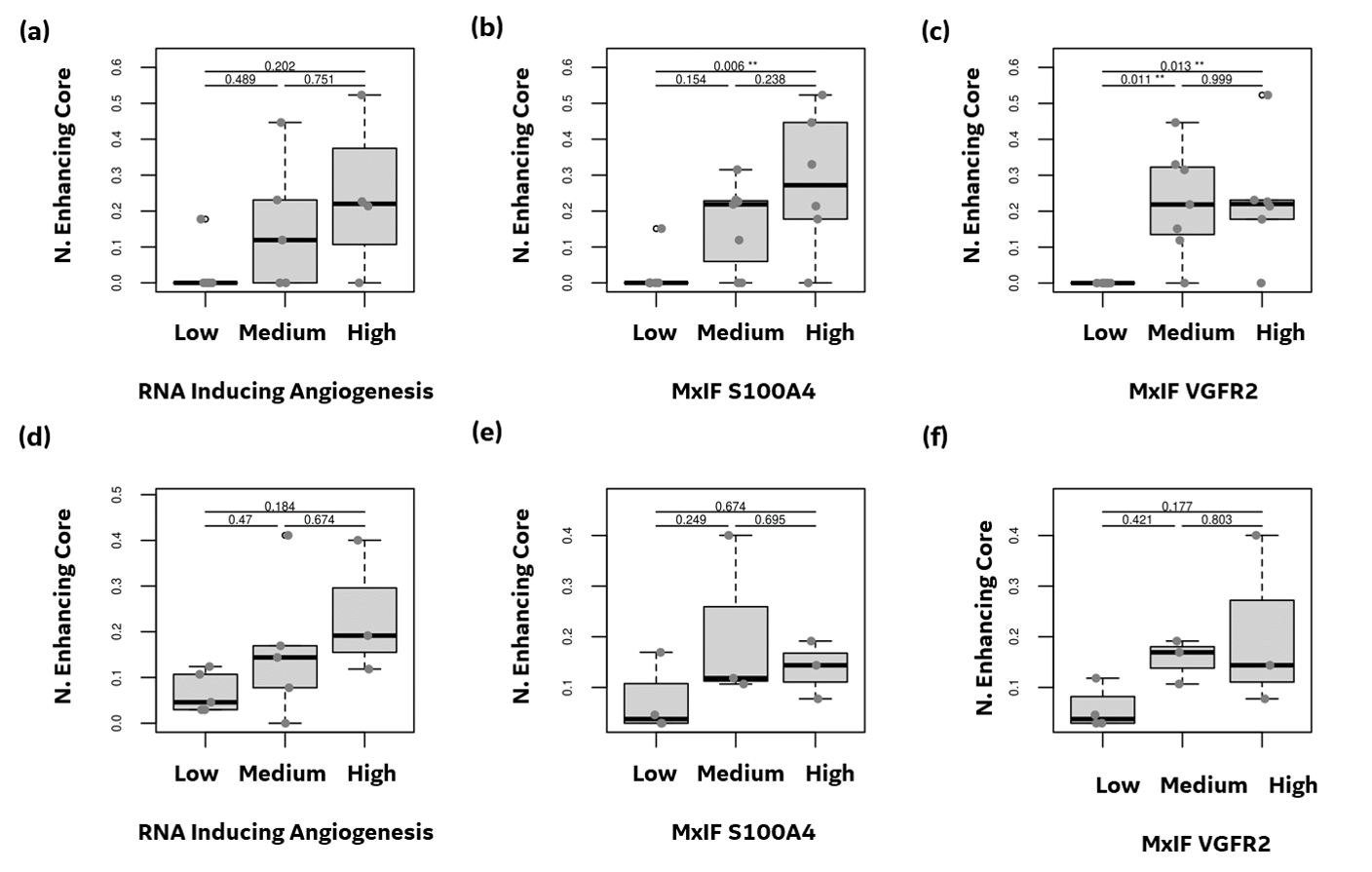

Supplement: S11 Fig — Correlation between Normalized enhancing core volume (derived from MRI) and Angiogenesis estimated from (a,d) RNA expression levels, and, based on multiplex immunofluorescence (MxIF) angiogenesis markers (b, e) S100A4 and (c,f) VEGFR2; (a-c) shows the plots on Cohort 1 (CW Glioma, treatment naive) while (d-f) show cohort 2 (UCSF, recurrent GBM). A progressive increasing trend may be observed in both cohorts when examining the normalized enhancing core volume for low, medium and high angiogenesis. The trends across the enhancement ratio are also conserved when comparing RNA with MxIF Angiogenesis. None of these comparisons reach statistical significance after multiple comparison correction using false discovery rate. (TIF) [file pone.0219724.s015.tif]
