# Supplementary material for: Multiscale, multimodal analysis of tumor heterogeneity in IDH1 mutant vs wild-type diffuse gliomas
Source: PLoS One. 2019 Dec 27;14(12):e0219724. doi: 10.1371/journal.pone.0219724 (PMC6934292; doi:10.1371/journal.pone.0219724)
Supplement: Attachment — Submitted filename: PLOS ONE comments.docx [file pone.0219724.s016.docx]

PLOS ONE

Tumor cell phenotype and heterogeneity differences in IDH1 mutant vs wild-type gliomas

(Reviewer Comments to the Author)

Based on my review, these results have not been published elsewhere (except Biorxiv preprint).

In this study, the authors have used multiple platforms to investigate phenotype and heterogeneity differences in IDH1 mutant vs wild-type gliomas. Some interesting findings are presented and the study represents an essential contribution to the field. The approach for developing multiscale and multi parametric data to study differences in glioma subtypes and the way to assemble the analysis is the convincing and novel part of the study.

The manuscript contains large number of figures and tables demanding a more thorough presentation and conformity. This will make it easier to keep a track on the story. There are some gaps in the development of the arguments and some of the details/discussion/explanations were difficult to find.

Following are the points to work on and need to be answered.

1) Experimental statistics are not performed to a technical standard and are not described in sufficient detail. There is not much information about the statistical analysis in the whole manuscript and the figures legends do not have any details about the statistics performed such as tests, p value (not everywhere), significance and the correction (if used). (for example figure 1, 6 and S6A and S11). The author should provide a section in materials and method for detailed statistical analysis and software used.
2) The authors should provide the gene/protein name of the lollipop bars in Figure 3B and 3E (where the other genes are unchanged or slightly changed) for transparency.
3) In the Figure 5A and B, where author is comparing the cell dive biomarker intensity with bulk mRNA expression, there is high contradiction in the FASN and GSK3b expression. The author should also explain or discuss about the difference in expression values coming from cell dive and mRNA seq data for FASN and GSK3B. Western blot should be performed to verify the difference in the expression level of FASN and GSK3B in IDHwt and IDHmt tumors.
4) Figure 3A(h) where FASN overlaid on DAPI, DAPI staining does not look convincing (surprisingly, not all the cells were positive for DAPI in 3A(h) while in figure 3D (h) the DAPI staining looks fine). Any explanation?
5) (in the provided excel sheet) It is mentioned that false staining means staining failed or not observed in any of the cores and this were excluded from the analysis. There is a possibility that particular proteins are expressed in that tissue type. Could this be a source of bias? Did authors use positive controls tissue sections in these situations and were lack of staining with antibodies and presence of protein validated?
6) The mRNA seq data analysis could give many other interesting findings. The authors should also show the expression of few genes for CD8, NK cells, GD T cells, microglia/macrophages and MDSC cells in IDHmt and IDHwt, as they are important shares of the brain tumor microenvironment and contribute up to 50% of the total population.
7) The title of the paper should be changed and should mention the use of multiscale workflow/technology in the study. Especially, because major part of this study is describing the method and new way of analysis.
8) The abstract could be more informative on the specific findings of the study.
9) On the line 689 it says weather, it should be whether.
