# Supplementary material for: Multiscale, multimodal analysis of tumor heterogeneity in IDH1 mutant vs wild-type diffuse gliomas
Source: PLoS One. 2019 Dec 27;14(12):e0219724. doi: 10.1371/journal.pone.0219724 (PMC6934292; doi:10.1371/journal.pone.0219724)
Supplement: Attachment — Submitted filename: Response to Critique.docx [file pone.0219724.s017.docx]

Michael E. Berens, Ph.D.

Deputy Director, TGen Research Resources

Division Director, Cancer & Cell Biology

Professor, Brain Tumor Unit

t (602) 343-8760

e mberens@tgen.org

October 4, 2019

PLOS ONE

**RE: “**Tumor cell phenotype and heterogeneity differences in IDH1 mutant vs wild-type gliomas” retitled as, “***Multiscale, multimodal analysis of tumor heterogeneity in IDH1 mutant vs wild-type diffuse gliomas***”

The critiques of the prior submission, as well as the authors’ responses/edits are noted below. A clean copy (manuscript.docx) as well as a red-lined version indicating changes to the manuscript (redlined_manuscript.docx) are submitted, as well as figures and tables, which were processed through the PACE tool as recommended on the PLoS ONE website, and given PACE Figure numbers as sown in the spreadsheet submitted.

1) Experimental statistics are not performed to a technical standard and are not described in sufficient detail. There is not much information about the statistical analysis in the whole manuscript and the figures legends do not have any details about the statistics performed such as tests, p value (not everywhere), significance and the correction (if used). (for example figure 1, 6 and S6A and S11). The author should provide a section in materials and method for detailed statistical analysis and software used.

>>> *Yes, this was an oversight on our part. A new section on statistical analysis methods has been added at the end of the methods section. Here we have included the statistical methods and cited R software for biomarker, cluster, MR feature and heterogeneity comparison between IDHmt vs wt patients and survival analysis. Extensive detail was already provided on the k-means clustering and spatial analysis methods.*

2) The authors should provide the gene/protein name of the lollipop bars in Figure 3B and 3E (where the other genes are unchanged or slightly changed) for transparency.

   >> >*Figure 3 has now been updated with all the protein names*
3) In the Figure 5A and B, where author is comparing the cell dive biomarker intensity with bulk mRNA expression, there is high contradiction in the FASN and GSK3b expression. The author should also explain or discuss about the difference in expression values coming from cell dive and mRNA seq data for FASN and GSK3B. Western blot should be performed to verify the difference in the expression level of FASN and GSK3B in IDHwt and IDHmt tumors.

>>> *Despite the differences for FASN, GSK3b and NCad, the concordance between the two technologies is notable for most other markers, particularly given the differences in sample amount and preservation (fixed vs frozen). Otherwise,* *directional discordance between mRNA and protein for any given gene is not an unanticipated finding, and on an individual gene level, is more discordant in tumor versus cognate normal tissue (Idit Kosti, Nishant Jain, Dvir Aran, Atul J. Butte & Marina Sirota. Cross-tissue Analysis of Gene and Protein Expression in Normal and Cancer Tissues.*

[*Scientific Reports volume 6, Article number: 24799 (2016*](https://www.nature.com/articles/srep24799)*). Underlying reasons for this discordance include variant turnover rate of the protein versus mRNA, translational efficiency due to ribosomal density and occupancy, RNA secondary structure, among others (*[*Correlation of mRNA and Protein in complex biological samples. 2009*](https://doi.org/10.1016/j.febslet.2009.10.036)*). Unfortunately it is not possible to conduct additional western analysis due to sample depletion. These points have also now been noted in the revised paper.*

4) Figure 3A(h) where FASN overlaid on DAPI, DAPI staining does not look convincing (surprisingly, not all the cells were positive for DAPI in 3A(h) while in figure 3D (h) the DAPI staining looks fine). Any explanation?

*>>>Since all images in the same group are set to same window/level in ImageJ, DAPI staining in 3A(h) is not readily apparent, however, the relative DAPI intensities between two images are different. Since these are two different patients the differences may be due to fixation conditions or age of the sample.*

5) (in the provided excel sheet) It is mentioned that false staining means staining failed or not observed in any of the cores and this were excluded from the analysis. There is a possibility that particular proteins are expressed in that tissue type. Could this be a source of bias? Did authors use positive controls tissue sections in these situations and were lack of staining with antibodies and presence of protein validated?

*>>>Yes, a control TMA containing both positive and negative controls used for antibody validations were included in the multiplexing study to confirm antibody performance (described in the methods section, lines 197 and 201). Additionally, “Control cores were included on all slides for glioma, prostate, melanoma, lung, breast cancer (two per cancer type) to verify antibody performance.” (line 183-184). Her2 was not expected to stain positive, nor cytokeratin and many of the other markers with negative staining were immune related, suggesting perhaps an immunosuppressive environment.*

6) The mRNA seq data analysis could give many other interesting findings. The authors should also show the expression of few genes for CD8, NK cells, GD T cells, microglia/macrophages and MDSC cells in IDHmt and IDHwt, as they are important shares of the brain tumor microenvironment and contribute up to 50% of the total population.

*>>> We agree the RNA Seq data could provide other findings. Our focus in this paper was to investigate the RNA and protein expression profiles and metrics at a pathway/hallmark level and compare those with MR features. However, immune evasion hallmark was not found to be a significantly correlated with MR features for either this cohort, nor in an earlier TCGA analysis of RNA and MR data from the Cancer Imaging Archive. Data will be made available for further analysis.*

7) The title of the paper should be changed and should mention the use of multiscale workflow/technology in the study. Especially, because major part of this study is describing the method and new way of analysis.

*>>> Appreciate the helpful suggestion, we have changed the title to: Multiscale, multimodal analysis of tumor heterogeneity in IDH1 mutant vs wild-type diffuse gliomas*

8) The abstract could be more informative on the specific findings of the study.

>>> The information content of the abstract now contains additional specific findings of the study

9) On the line 689 it says weather, it should be whether.

*>> Changed*

Thank you for your consideration.

Sincerely,

Michael E. Berens, Ph.D.
